# Recent advances in nucleotide analogue-based techniques for tracking dividing stem cells: An overview

**DOI:** 10.1016/j.jbc.2021.101345

**Published:** 2021-10-28

**Authors:** Georgy M. Solius, Dmitry I. Maltsev, Vsevolod V. Belousov, Oleg V. Podgorny

**Affiliations:** 1 Shemyakin-Ovchinnikov Institute of Bioorganic Chemistry, Russian Academy of Sciences, Moscow, Russia; 2 Federal Center of Brain Research and Neurotechnologies, Federal Medical Biological Agency, Moscow, Russia; 3 Center for Precision Genome Editing and Genetic Technologies for Biomedicine, Pirogov Russian National Research Medical University, Moscow, Russia

**Keywords:** thymidine analogues, stem cells, proliferation, BrdU (5-bromo-2′-deoxyuridine), EdU (5-ethynyl-2′-deoxyuridine), immunohistochemistry, click chemistry, AmdU, (azidomethyl)-2′-deoxyuridine, BrdU, 5-bromo-2′-deoxyuridine, CldU, 5-chloro-2′-deoxyuridine, EdU, 5-ethynyl-2′-deoxyuridine, F-ara-EdU, (2′S)-2′-deoxy-2′-fluoro-5-ethynyluridine, IdU, 5-iodo-2′-deoxyuridine, LRC, label-retaining cell, MIMS, multi-isotope imaging mass spectrometry, VdU, 5-vinyl-2′-deoxyuridine

## Abstract

Detection of thymidine analogues after their incorporation into replicating DNA represents a powerful tool for the study of cellular DNA synthesis, progression through the cell cycle, cell proliferation kinetics, chronology of cell division, and cell fate determination. Recent advances in the concurrent detection of multiple such analogues offer new avenues for the investigation of unknown features of these vital cellular processes. Combined with quantitative analysis, temporal discrimination of multiple labels enables elucidation of various aspects of stem cell life cycle *in situ*, such as division modes, differentiation, maintenance, and elimination. Data obtained from such experiments are critically important for creating descriptive models of tissue histogenesis and renewal in embryonic development and adult life. Despite the wide use of thymidine analogues in stem cell research, there are a number of caveats to consider for obtaining valid and reliable labeling results when marking replicating DNA with nucleotide analogues. Therefore, in this review, we describe critical points regarding dosage, delivery, and detection of nucleotide analogues in the context of single and multiple labeling, outline labeling schemes based on pulse-chase, cumulative and multilabel marking of replicating DNA for revealing stem cell proliferative behaviors, and determining cell cycle parameters, and discuss preconditions and pitfalls in conducting such experiments. The information presented in our review is important for rational design of experiments on tracking dividing stem cells by marking replicating DNA with thymidine analogues.

Somatic stem cells are cells able to self-renew and to produce progeny that undergo differentiation into functional organ- or tissue-specific cells. Somatic stem cells support continuous physiological organ or tissue renewal and/or regeneration of injured organs or tissues. Disturbance of somatic stem cell functioning is thought to underlie many degenerative diseases in humans, and *in situ* stem cell activation and transplantation into injured organs are considered as promising therapeutic strategies to delay or resist the onset of diverse degenerative disorders.

Stem cells undergo mitotic division to realize their functions within an organism. Delivery and subsequent detection of thymidine analogues that incorporate into replicating DNA during the S-phase of the cell cycle are one of the basic approaches for tracing the fate of dividing stem cells and their progeny in diverse *in vitro* and *in vivo* systems. Several comprehensive reviews have already described the application of nucleotide analogues for marking replicating DNA (1, 2, 3, 4, 5). These reviews addressed questions regarding the technical aspects of nucleotide analogue detection using antibodies or bioorthogonal chemical reactions, approaches for double S-phase labeling, applications of modified nucleotides for stem cell research, investigation of spatiotemporal features of DNA replication, multiparametric cell cycle analysis by flow cytometry, and labeling of living cells. In this review, we will focus on (i) critical points regarding delivery, dosage, and detection of nucleotide analogues for single- and multilabel marking of replicating DNA, (ii) applications of pulse-chase and cumulative labeling schemes and their combinations for determining cell cycle parameters and for revealing specific modes of cell cycle behavior, such as re-entering and exiting the cell cycle, (iii) caveats to consider when applying labeling with modified nucleotides, and (iv) the most recent advances in detection of replicating DNA. These topics are largely absent in previous reviews.

## Delivery and detection of nucleotide analogues

### A brief overview on marking replicating DNA

Replication of genetic material is a key process underlying cell division. It is essential for creating multicellularity and multiplication of all organisms. A cell replicates its DNA when passing through the S-phase of the cell cycle. Tagging replicating DNA enables nuclei of dividing cells and their progenies to be marked due to ability of the tag to remain within the replicated DNA for prolonged periods.

Labeling replicating DNA with the radioactive nucleoside ^3^H-thymidine, which is a precursor of one of the four chemical building blocks of DNA, and its detection by autoradiography was initially introduced by Taylor *et al*. in 1957 (6). In their work they used autoradiographic analysis of chromosome preparations from *Vicia faba* seedlings treated with ^3^H-thymidine and revealed that only one of the two sister chromatids in each chromosome was radioactive in the cells of the roots collected after the second replication cycle. Thus, during replication, daughter chromosomes receive an original and a new strand. This observation supported the semiconservative replication model. Later, the delivery of ^3^H-thymidine and another radioactive nucleoside, ^14^C-thymidine, with subsequent autoradiographic detection revealed features and mechanisms of DNA replication in pro- and eukaryotic cells, such as unwinding of the double helix, formation of the replication fork, spatial patterning of DNA replication, and creation of the lagging DNA strand through intermittent synthesis of Okazaki fragments (reviewed in (1, 2)). By tracing dividing cells and their progeny by autoradiographic detection, ^3^H-thymidine was widely employed in developmental biology, regenerative biology, and stem cell research. For instance, this approach enabled birth dating of neurons within different cortical layers during corticogenesis in mammals (7), identification of satellite cells as muscle stem cells and a cellular source for muscle regeneration (8), and discovery of the continuous production of new neurons in the walls of the lateral ventricles and the hippocampus in the adult mammalian brain (9, 10).

^3^H-thymidine is used for marking replicating DNA because, unlike the other nucleosides, ^3^H-thymidine is a precursor of DNA but is not involved in RNA synthesis (11). The major disadvantages of ^3^H-thymidine are handling of a radioactive substance and the use of the time-consuming autoradiography method for detection. Detection of 5-bromo-2′-deoxyuridine (BrdU) (Table 1), a synthetic nucleoside analogue of thymidine, is an alternative technique for the determination of DNA replication and has overcome these disadvantages (12, 13). BrdU incorporated into DNA is recognized by a specific polyclonal or monoclonal antibody produced against bromouridine or iododeoxyuridine complexed to a carrier protein such as bovine serum albumin. The ability to combine BrdU labeling with the detection of cell-type-specific markers *via* specific antibody staining or reporter gene expression has become a gold standard for studying cell division and differentiation, which are major cellular processes underlying development in multicellular organisms and tissue renewal and regeneration in adulthood.

**Table 1:** Summary on modified nucleotides

Nucleotide analogue	M.W. (g/mol)	Doses used for intraperitoneal injections in rodents	DNA denature	Antibody detection	Chemical detection	Solubility in water-based solvents (maximal concentrations that have been reported), mg/ml	Relative cytotoxicity
BrdU	307.1	Regular dose: 50–100 mg/kg (52, 68); the saturating dose in mice: 150 mg/kg (40), the saturating dose in rats: 300 mg/kg (61)	Necessary	Yes	Optimized Suzuki–Miyaura reaction (DNA denature is unnecessary) (27)	15 (160) or 20 (with the addition of 0.007N NaOH) (82)	++
CldU	265.65	Regular dose: ∼40–128 mg/kg. Saturating dose has not been determined yet (41, 52, 54, 113).	Necessary	Yes. Rat monoclonal anti-BrdU antibody (clone BU1/75) specifically recognizes CldU	Not applied	6.4 (41) or 10 (with the addition of 0.007N NaOH) (54, 82)	Not yet determined
IdU	354.1	Regular dose: ∼60–173 mg/kg. Saturating dose has not been determined yet (41, 52, 54, 113)	Necessary	Yes. Mouse monoclonal anti-BrdU antibody (clone B44) specifically recognizes IdU	Not applied	2.47 (41) or 10 (with the addition of 0.007N NaOH) (54)	Not yet determined
EdU	252.23	Regular dose: 50 mg/kg. Saturating dose in mice: approx. 100 mg/kg (62)	Unnecessary	Yes. All anti-BrdU antibodies recognize EdU after DNA denature, excluding mouse monoclonal anti-BrdU antibody clone MoBU1.	Cu(I)-catalyzed [3 + 2] cycloaddition reaction (22)	6.15 (41)	++++
F-ara-EdU	270.21	Not yet determined	Unnecessary	Not yet determined	Cu(I)-catalyzed [3 + 2] cycloaddition reaction (24)	Not yet determined	+
AmdU	283.24	Not yet determined	Unnecessary	Not yet determined	Cu(I)-catalyzed [3 + 2] cycloaddition reaction (25)	Not yet determined	+++
VdU	254.24	Not yet determined	Necessary	Not yet determined	Alkene–tetrazine ligation reaction (26)	Not yet determined	+++

Due to their nonoverlapping detection techniques, BrdU and ^3^H-thymidine can be combined in one sample allowing for temporal discrimination of DNA synthesis in dividing cells to reveal their progression through the cell cycle (14, 15, 16, 17, 18, 19). However, the detection of truly double labeled nuclei in tissue sections using immunohistochemistry and autoradiography may be compromised, because anti-BrdU antibodies stain cell nuclei and the radioactive label causes formation of silver grains in the photographic emulsion covering the tissue section surface. Therefore, if the nuclei of two cells overlap when we observe a tissue section through the microscope, we are unable to judge unequivocally whether we see a truly double labeled cell or two cells that have incorporated different labels. Application of the halogenated thymidine analogues 5-chloro-2′-deoxyuridine (CldU) and 5-iodo-2′-deoxyuridine (IdU) that resemble BrdU in their ability to tag replicating DNA (Table 1) and extension of the antibody panel for their recognition have led to the development of a method for concurrent detection of two distinct labels within a sample, allowing for birth dating of various cohorts of cells (20).

The major drawback of detection of halogenated thymidine analogues using antibodies is the necessity of DNA denaturation, usually in a 0.5 N–4 N hydrochloric acid solution, prior to sample processing, because of the inaccessibility of the BrdU epitope within complimentary paired bases. The DNA denaturation procedure erodes cell and tissue components limiting the use of various concurrent molecular assays. In particular, acidic DNA denaturation prior to BrdU detection by antibodies leads to poor staining of samples with widely used nuclear dyes such as propidium iodide, DAPI, or Hoechst stains. A variety of approaches were proposed to overcome this drawback. These include (i) treatment with sodium hydroxide, which disrupts the DNA structure *via* deprotonation of the nucleobases, (ii) incubation with various nucleases (for instance, exonuclease III) or nuclease mixtures to generate single-stranded regions, in which the antibody is able to bind to BrdU, (iii) exposure to monovalent copper ions, which, in the presence of oxygen, oxidizes deoxyribose moieties, producing DNA breaks, (iv) ultraviolet light photolysis, and (v) heating (1, 12, 21).

**Figure 1:**
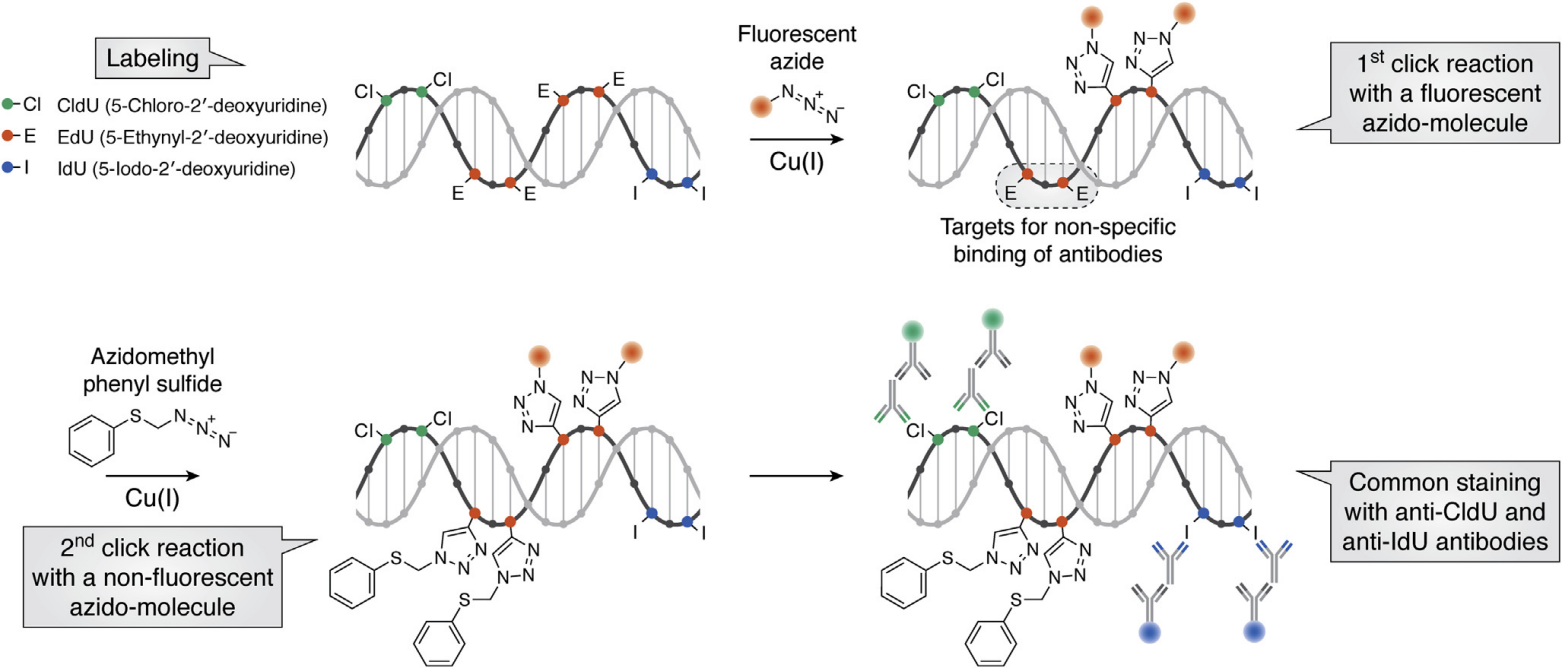
**Key staining stages for the concurrent detection of three nucleotide analogues: CldU, IdU, and EdU.** Here, the nonfluorogenic second click reaction is necessary for blocking nonspecific binding of anti-CldU and anti-IdU antibodies to residual EdU, which has not reacted with a fluorescent azide.

A method for chemical detection of another synthetic nucleoside analogue of thymidine, 5-ethynyl-2′-deoxyuridine (EdU) (Table 1), has been created (22). The method is based on the incorporation of EdU into replicating DNA and its subsequent detection by the covalent coupling of a fluorescent azide to a terminal alkyne group through a Cu(I)-catalyzed [3 + 2] cycloaddition reaction, frequently called a ‘‘click’’ reaction (see Fig. 1 in (22)). This method does not require denaturation of DNA and, therefore, lacks the major drawback related to the use of halogenated thymidine analogues. EdU labeling followed by detection using the click reaction preserves the integrity of cell and tissue components allowing for a variety of concurrent molecular assays to be performed. The click reaction is a bioorthogonal chemical reaction, a type of reaction that does not interfere with biochemical reactions occurring in live cells (23).

The latest advances in creating strategies for probing biological macromolecules with a synthetic tag containing a bioorthogonal functional group have extended the toolset for labeling replicating DNA. Reported novel nucleotide analogues include (2′S)-2′-deoxy-2′-fluoro-5-ethynyluridine (F-ara-EdU), 5-(azidomethyl)-2′-deoxyuridine (AmdU), and 5-vinyl-2′-deoxyuridine (VdU) (Table 1). VdU is detected by a nonoverlapping bioorthogonal chemical reaction (an alkene–tetrazine ligation reaction). Therefore, it can be combined with other nucleotide analogues that are detected *via* the azide–alkyne click reaction and even to halogenated nucleotide analogues to produce multilabel marking of replicating DNA (24, 25, 26). Finally, a very recent study (27) reported a novel strategy that has revolutionized identification of BrdU incorporated into DNA. This novel strategy utilizes the Suzuki–Miyaura reaction to detect BrdU with fluorescent boronic acid probes instead of the traditionally used anti-BrdU antibodies.

Besides simple tracing of dividing cells and their progenies, several labeling schemes that combine pulse-chase and cumulative labeling with multilabel marking of replicating DNA have been developed for revealing specific modes of stem cell proliferative behavior and for evaluating key cell cycle parameters (Table 2). Application of such labeling schemes (see below) enables creating descriptive models for tissue histogenesis and renewal and stem cell maintenance, differentiation, and elimination.

**Table 2:** Summary on assays that can be performed using single, double, and triple S-phase labeling with modified nucleotides

Assay	Single S-phase labeling	Double S-phase labeling	Triple S-phase labeling
Proliferation assay	Pulse-chase labeling with the chase period less than the average cell cycle length (40, 64)	-	-
Retrospective birth-dating of cells and tracing cell fate	Pulse-chase labeling with the extended chase period (7, 45, 90)	Pulse-chase labeling with two temporally discriminated labels (20, 54)	Pulse-chase labeling with three temporally discriminated labels (88)
Tracing fate of post-mitotic cells	-	Window labeling (17)	-
Label-retaining cells	Cumulative labeling (161)	-	-
Determination of the S-phase and the cell cycle durations	Cumulative labeling (100, 101, 102, 103, 104, 105, 106, 107, 108, 109, 110) and percent labeled mitoses method (100, 110)	Pulse-chase labeling with two labels delivered at variable time intervals (64, 112, 113)	Pulse-chase labeling with three temporally discriminated labels (41)
Determination of the G_1_- and G_2_-phase durations	Percent labeled mitoses method (100, 110)	-	-
Estimation of the size of proliferative population	Cumulative labeling (100, 101, 102, 103, 104, 105, 106, 107, 108, 109, 110)	-	-
Proliferating and quiescent subpopulations of daughter cells	-	A combination of pulse-chase and cumulative labeling (14, 16, 117)	Pulse-chase labeling with three temporally discriminated labels (41)

### Delivery and dosage of nucleotide analogues

To mark replicating DNA in cell cultures, cells undergo treatment with a culture medium supplemented with a thymidine analogue at micromolar concentrations (usually 10–20 μM) (28, 29, 30, 31, 32). Larvae and adults of marine invertebrates (sea urchins, sponges, flatworms, etc.) are considered permeable for most pharmacological agents. Therefore, to mark replicating DNA in these species, nucleotide analogues are dissolved at micro- and millimolar concentrations in the ambient seawater they are maintained in (33, 34, 35). To label replicating DNA in fish (zebrafish (*Danio rerio*)) or frogs (*Xenopus laevis*), larvae and even adults can also be bathed in ambient water supplemented with millimolar concentrations of a nucleotide analogue (36, 37, 38, 39).

Thymidine analogues can be delivered into most vertebrate species through intraperitoneal, intravenous, or intramuscular injections, drinking water, and osmotic minipumps. Intraperitoneal injection is the easiest way to deliver a nucleotide analogue. Nucleotide analogues resemble natural thymidine; therefore they readily absorb into the blood stream after being injected intraperitoneally, spread broadly in the body through the blood circulation system, and penetrate virtually all organs and tissues of an organism, including those separated by barriers (brain, testis, placenta). Intraperitoneal injections are usually used in small animals, such as rodents (14, 16, 20, 40, 41), rabbits (42), small fish such as zebrafish (43), and frogs (44). Larger animals, such as monkeys, canines, or sheep, usually receive intravenous (45, 46, 47) or intramuscular injections (48) of a thymidine analogue.

Treatment with a thymidine analogue (usually BrdU) dissolved in drinking water is frequently used in rodent studies and is employed when long-term labeling of dividing cells is necessary. The concentration of BrdU in drinking water is typically 0.8 to 1 mg/ml (49, 50, 51, 52, 53, 54, 55). To overcome aversion to the taste of BrdU and to enhance intake of BrdU containing water to elevate labeling of dividing cells, drinking water is frequently supplemented with sucrose or orange juice (50, 56). This route of a nucleotide analogue delivery is used when distress evoked by daily intraperitoneal injections perturbs the resultant experimental data, or high animal mortality is observed. However, due to the circadian dependence of water intake, treatment with BrdU dissolved in drinking water marks different numbers of dividing cells during the light and dark phases of the day (50). The observed inaccuracy of BrdU labeling by delivery through drinking water may serve as source of artifacts that can in turn lead to misinterpretation of experimental data.

Subcutaneous implantation of osmotic minipumps charged with a thymidine analogue seems to be the most reliable method when long-term marking of replicating DNA is necessary (50). Commercially available osmotic minipumps provide a constant rate of the nucleotide delivery over a time interval of up to 28 days (51, 57, 58, 59, 60).

Independently from the route of delivery, the thymidine analogue dosage is of great importance when labeled cells are evaluated quantitatively and comparison between the experimental and control groups is necessary. We must ensure that changes in the number of labeled cells originate from alterations in proliferation, not from alterations in the nucleotide analogue uptake. A saturating dose, which is defined as the dose of a nucleotide analogue necessary to label most cells in the S-phase, satisfies this condition. The saturating dose of a nucleotide analogue depends on the species used, the organ studied, and the life stage analyzed. Theoretically, the saturating dose must be determined for each individual experimental condition. An accurate determination of saturating doses for BrdU and EdU has been reported for dividing cells in the hippocampal dentate gyrus of adult rodents (40, 61, 62) and the walls of the lateral ventricles of an adult male songbird zebra finch (*Taeniopygia guttata*) (63). Interest in the hippocampal dentate gyrus in regard to determining the saturating dose of a nucleotide analogue is due to the following. The hippocampal dentate gyrus is a spatially limited structure. In the dentate gyrus, dividing cells do not extensively migrate. They do not form very dense clusters, allowing for easy discrimination and counting of individual labeled nuclei using optical microscopy. Therefore, quantitative evaluations of proliferating cells in the whole dentate gyrus of animals of the same age are in a good agreement between many research groups (40, 41, 62, 64, 65).

**Figure 2:**
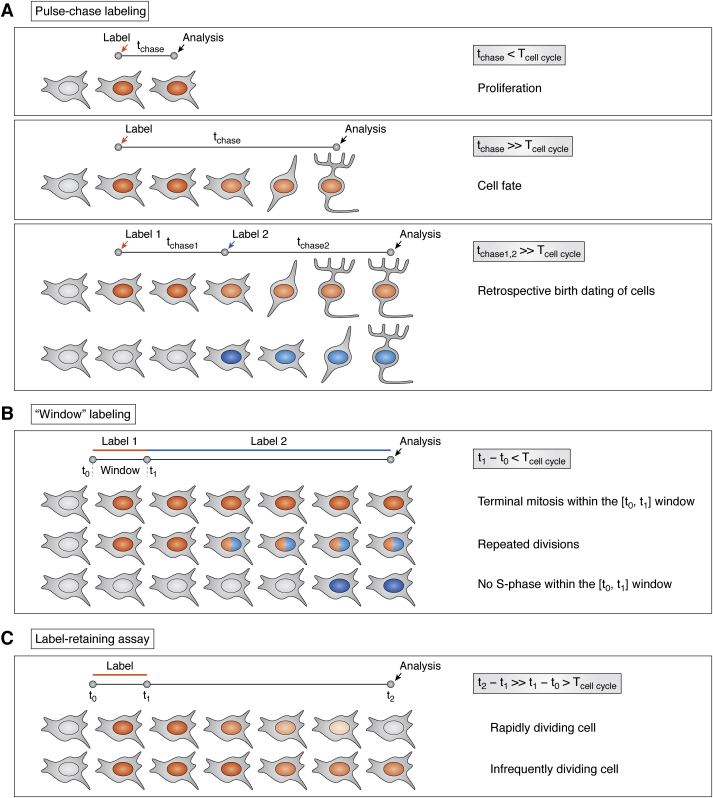
**Major labeling schemes used to reveal the fate of dividing cells.***A*, pulse-chase labeling. *B*, window labeling. *C*, label retaining assay.

To evaluate the saturating dose practically, animals separated into several experimental groups receive single intraperitoneal injections of various doses of a nucleotide analogue. The doses tested are usually in the range of 10 to 600 mg/kg body weight. Then, numbers of labeled cells are counted, and the dependence of cell counts on thymidine analogue dose is determined. Typically, the dependence is represented by an initial raise in the number of labeled cells along with the increase in the thymidine analogue dose, followed by a plateau where dose elevation does not increase the number of labeled cells (40, 61, 63, 66). The point where the dependence achieves a plateau indicates the saturating dose (see, for instance, Fig. 1*B* in (40) or Fig. 2 in (62)). Current estimations of the saturating dose of BrdU determined by quantitation of labeled cells in the hippocampal dentate gyrus after a single intraperitoneal delivery are 150 mg/kg body weight in mice (40) and 200 mg/kg (66) or 300 mg/kg (61) body weight in rats. Similarly, a very recent study (63) determined 50 mg/kg body weight as the saturating dose of BrdU for labeling dividing cells residing in the walls of the lateral ventricles of a songbird zebra finch. Lower doses result in underestimation of the proliferating cell number, whereas higher doses provide the same number of the labeled cells as the saturating dose.

Similarly, another study (67) reported determination of the BrdU saturating dose for the optimal labeling of dividing cells in the rat forestomach. The thymidine analogue was delivered continuously for 2 days through the subcutaneous implementation of flat-faced cylindrical matrices containing BrdU. A 200 mg dose (or on average 540 mg/kg body weight for 2 days) was found to label most dividing cells in the rat forestomach. The saturating dose of the rest of the halogenated nucleotide analogues remains to be determined. Although the saturating doses of BrdU have been accurately determined, most studies reported the use of the standard dose of BrdU, 50 to 100 mg/kg body weight in rodents (Table 1) (52, 68, 69, 70, 71). The doses within this range enable labeling of 60 to 90% of the proliferating cells that would be detected by a single delivery of the saturating dose. Accurate measurements have validated partial labeling by the standard doses for quantitative analysis and comparisons between experimental groups (40, 61, 72).

50 mg/kg and 41 mg/kg body weight doses of EdU were demonstrated to provide labeling at near-saturation level in terms of labeled cell numbers in the mouse dentate gyrus (62) (Table 1) and the walls of the lateral ventricles of a songbird zebra finch (63), respectively. The EdU dose 50 mg/kg is equimolar to the BrdU dose 61 mg/kg of body weight that labels approximately 70% of proliferating cells in the mouse dentate gyrus (40). This difference in the labeling level between BrdU and EdU seems to originate from the method of detection rather than from individual distinctions in labeling capacity of the thymidine analogues. BrdU was detected using immunohistochemistry combined with DAB (3,3′-Diaminobenzidine)-staining (40), whereas EdU was detected using the fluorogenic click reaction. Even suggesting equal sensitivity of both detection methods, the transmitted light microscopy required for observing DAB-stained samples provides lower contrast than fluorescent microscopy. There is a high risk of skipping slightly labeled nuclei when counting cells in DAB-stained samples. Therefore, underestimation of the proliferating cell number observed in DAB-stained samples can originate from quantification of labeled cells using transmitted light microscopy. To fill this gap, it is necessary to reevaluate the BrdU saturating dose using a fluorescently tagged antibody.

In sum, the observations mentioned above exhibit that dosage of a nucleotide analogue is a challenging problem, and preliminary tests are strongly recommended to elucidate behavior of the label under specific experimental circumstances. Importantly, dosage of a nucleotide analogue is also a compromise between labeling efficiency and adverse effects, such as overall cytotoxicity and cell cycle arrest (see below).

### Detection of nucleotide analogues by antibodies and click reaction

Several excellent reviews and detailed protocols have already described staining procedures for revealing thymidine analogues in diverse biological samples (1, 3, 22, 28, 73, 74, 75, 76). Here, we will delineate a workflow and briefly discuss principal stages for the detection of the most frequently used nucleotide analogues using antibodies and the click reaction.

Independently of biological sample type, the halogenated nucleotide analogues (BrdU, CldU, and IdU) necessitate DNA denaturation prior antibody detection (Table 1). The most frequently used method of DNA denaturation is the immersion of a sample into a 0.5 N–4 N hydrochloric acid solution. Solution temperature (usually 37 °C) and immersion time (usually 10–60 min) can be adjusted to obtain desirable staining quality. The drawbacks of acidic DNA denaturing and ways to bypass these drawbacks have been mentioned above (see also (1, 21)). To neutralize hydrochloric acid, samples undergo one or several rounds of treatment with 0.1 M borate (pH 8.5) at room temperature (77, 78, 79). This procedure is desirable when processing whole organisms or tissue sections because it partially reverses the wrinkling and shrinking of the sample evoked by hydrochloric acid exposure.

Revealing halogenated nucleotide analogues incorporated into DNA employs standard immunohistochemical (DAB) or immunofluorescent (a fluorescent tag) staining procedures, which include permeabilization, blocking nonspecific secondary antibody labeling, exposure to the primary and secondary antibodies, and signal amplification steps. Description of these procedures can be found elsewhere (80, 81). To detect halogenated nucleotide analogues, numerous antibodies produced in various hosts are available from diverse commercial sources. This provides great flexibility in the concurrent detection of halogenated nucleotide analogues and various biomolecules in the same specimen using different labeling techniques. However, the level of BrdU labeling has also been found to strongly depend on the primary antibody used for BrdU detection both in terms of labeled cell numbers and in terms of fluorescence intensity (brightness of labeled cells) (28, 82). As large as a twofold difference in the number of BrdU labeled cells in the hippocampal dentate gyrus has been observed with antibodies originating from distinct commercial sources (82). An even more drastic variability in the intensity of the fluorescent signal was found after applying different primary anti-BrdU antibodies to HeLa cells treated with BrdU (28). Both reports have unambiguously demonstrated that primary anti-BrdU antibodies originating from distinct sources are not equally sensitive to nucleotide analogues. Most primary anti-BrdU antibodies have been found to cross-react to EdU, excluding mouse monoclonal anti-BrdU antibody (clone MoBU1) (28). Mouse monoclonal anti-BrdU antibody (clone B44), which is sensitive to BrdU and IdU, exhibited reduced cross-reactivity to CldU, whereas rat monoclonal anti-BrdU antibody (clone BU1/75) reacts with both BrdU and CldU and is insensitive to IdU (82).

EdU incorporated into DNA is detected by the covalent binding of a fluorescent azide to a terminal alkyne group through a Cu(I)-catalyzed [3 + 2] cycloaddition reaction (22, 62, 83, 84). The detection of EdU is much easier than the detection of halogenated nucleotide analogues. Permeabilization is the only procedure required prior to EdU detection by the click reaction. The covalent binding of a fluorescent azide to a terminal alkyne group in the EdU residue requires the presence of monovalent copper ions. The click reaction mixture is supplemented with a reducing agent, usually ascorbic acid or its sodium salt (+)-Sodium L-ascorbate to obtain monovalent copper ions from divalent copper ions, which are usually obtained by dissolving copper-containing salts such as CuSO_4_ (22, 41, 84). Interestingly, a Cu(I)-catalyzed [3 + 2] cycloaddition reaction can be performed without fixation, allowing for staining of live EdU-labeled cells both *in vitro* and *in vivo* (22). To this end, live cells or acutely prepared tissue samples pretreated with EdU are administrated with a staining solution supplemented with CuSO_4_, ascorbic acid, and cell-membrane-permeable tetramethylrhodamine azide. However, Cu(I) ions are highly toxic, and therefore the cells do not survive staining. Although live microscopy of EdU-labeled and azide-stained cells is of limited utility, it may be useful in those cases where subsequent molecular assays are not compatible with formaldehyde fixation and/or permeabilization of cell membranes, or when they must be performed without removal of the samples from the microscope stage to overlap other experimental readouts with the EdU labeling (22).

Detection of both halogenated nucleotide analogues and EdU is accompanied with the exposure of a biological specimen to highly chemically active substances such as hydrochloric acid or monovalent copper ions, which can affect the biochemical properties of macromolecules and the integrity of cellular components. Therefore, if the concurrent detection of other macromolecules within the same specimen is necessary, the protocol for thymidine analogue detection must be adjusted for the specific experiment to diminish the negative effects of the staining procedures on the other components to be detected.

### Critical points for the concurrent detection of several nucleotide analogues within the same sample

In the brief overview on marking replicating DNA, we have already mentioned that detection of truly double labeled nuclei may be compromised when employing the double labeling technique using BrdU and ^3^H-thymidine. Moreover, this double labeling technique is not compatible with current fluorescence microscopy, such as confocal or light-sheet microscopy. This limitation narrows the range of research issues that can be addressed by the double nucleotide labeling with BrdU and ^3^H-thymidine. Therefore, we will focus here on the current double and triple labeling techniques that exploit antibodies and bioorthogonal chemical reactions for detection of nucleotide analogues.

Double labeling using CldU and IdU was initially presented in 1992 (85, 86). This double labeling method is based on the different sensitivity of two monoclonal antibodies against BrdU. One antibody, rat anti-BrdU antibody clone BU1/75, recognizes both BrdU and CldU, but exhibits low binding to IdU. The other antibody, mouse anti-BrdU antibody clone B44, recognizes BrdU and IdU, while displaying low binding to CldU. Procedures such as sequential application of the primary antibodies, washing in Tris-buffered saline with a high salt concentration, and determination of appropriate antibody dilutions have been proposed to remove residual cross-reactivity of rat anti-BrdU antibody clone BU1/75 to IdU and mouse anti-BrdU antibody clone B44 to CldU in both *in vitro* and *in vivo* examinations (20, 41, 85, 86, 87). Detection of a noncognate nucleotide analogue by these antibodies can also originate from nonspecific binding of the secondary antibodies that imperfectly discriminate between mouse and rat immunoglobulins. Therefore, the use of the secondary antibodies that have been additionally cross-adsorbed to the respective immunoglobulins is strongly recommended (41). Optionally, primary antibodies conjugated with fluorescent tags of different colors can be used to increase the specificity of CldU and IdU detection (88).

Detection of yet another pair, BrdU and EdU, lacks any residual cross talk, thus enabling unequivocal discrimination between truly double and single labeled cells. Initially, ten different anti-BrdU antibodies from various commercial sources were examined for their reactivity to EdU (28). It was found that most anti-BrdU antibodies bind to EdU residues in the DNA except the mouse monoclonal anti-BrdU antibody clone MoBU1 (28). Therefore, the delivery of EdU and BrdU followed by their detection *via* the click reaction and the application of the mouse monoclonal anti-BrdU antibody clone MoBU1 is a reliable double labeling method. Interestingly, when cultured cells labeled with EdU undergo staining with anti-BrdU antibodies, the signal of most anti-BrdU antibodies (except clone MoBU1) is not completely removed even by prolonged click reaction or the click reaction with an elevated fluorescent azide concentration (28, 41). Then, several nonfluorescent azides were examined to determine if they are able to inhibit anti-BrdU binding when applied after the fluorogenic click reaction (28). Among the examined nonfluorescent azides, azidomethyl phenyl sulfide was found to completely remove the signal of anti-BrdU antibodies at a low concentration of 2 mM. Therefore, the application of the click reaction with azidomethyl phenyl sulfide (the second click reaction) after the fluorogenic click reaction (the first click reaction) and prior to the anti-BrdU antibody staining is a reliable alternative approach for the concurrent detection of EdU and BrdU.

Recently, we reported triple S-phase labeling of dividing cells *in vivo* and the protocol for concurrent detection of CldU, IdU, and EdU (41, 89). The reported protocol combines the detection of CldU and IdU *via* application of the rat anti-BrdU antibody clone BU1/75 and the mouse anti-BrdU antibody clone B44 with inhibition of antibody binding to EdU using a second click reaction with azidomethyl phenyl sulfide (Fig. 1). In our study, we validated the method for triple S-phase labeling qualitatively and quantitatively. The complete protocol for the detection of the three nucleotide analogues was applied to brain sections of mice that received a single injection of an individual nucleotide analogue. Microscopic analysis confirmed that the complete protocol specifically detects individual nucleotide analogues in these mice without any cross-reactivity. Quantitative analysis of labeled cells in the hippocampi of mice that received sequential injections of all three thymidine analogues at fixed time intervals revealed full agreement with the predicted parameters of triple labeling in a system with known cell cycle kinetics. Moreover, this quantitative analysis also confirmed equality in the labeling capacity of the examined thymidine analogues when delivered at equimolar doses. The importance of equimolar delivery was indicated for maintenance of the quantitative relationship between cell populations incorporating two labels at different time points (20). Triple S-phase labeling has recently been applied to verify the model of slow depletion of chondroprogenitors by direct recruitment during longitudinal bone growth (88).

Another study reported a novel approach for the concurrent detection of three other thymidine analogues, F-ara-EdU, VdU, and BrdU, using two nonoverlapped bioorthogonal chemical reactions and antibodies against BrdU (26). Applications of the nucleotide analogues that are detected *via* bioorthogonal chemical reactions are described below.

## Labeling schemes and respective readouts for revealing stem cell proliferative behaviors and fates

### Pulse-chase labeling

“Pulse labeling” (or “a pulse dose”) can be defined as labeling dividing cells when a thymidine analogue is available for incorporation into replicating DNA within a time interval that is shorter than the average cell cycle duration of a given cell population. On average, the cell cycle of most eukaryotic cells lasts for several hours. Therefore, treating cultured cells, or bathing larvae or adult organisms with a thymidine analogue for several hours or less may be considered as pulse labeling. A single intraperitoneal or intravenous injection of a thymidine analogue may also be considered as pulse labeling because of its transient availability in the blood (the bioavailability of modified nucleotides is described below). Pulse labeling is followed by a “chase” period when the nucleotide analogue is not delivered. The chase period in this labeling scheme may be varied in a wide range, from several minutes to months and even years, depending on purposes of the study (40, 45, 90). Thus, pulse-chase labeling enables dividing cells that were passing the S-phase of the cell cycle at the time point when a single nucleotide analogue was delivered, to be traced (7, 9) (Fig. 2*A*). If the chase period after pulse labeling is less than the average cell cycle length of a given cell population, cells that have incorporated the label are considered to be dividing cells (cells passing the cell cycle), and quantification of these cells allows for estimation of proliferative activity (proliferation assay). Extension of the chase period enables the progeny of cells that were passing the S-phase of the cell cycle at the time point when a single nucleotide analogue was delivered to be tracked. Tracking the progeny of dividing cells combined with identifying differentiation markers allows the fates of cells generated at a certain time to be determined. Therefore, this labeling scheme is extensively used in developmental biology and stem cell research. Pulse-chase labeling with two and even three nucleotide analogue species allows for the discrimination of cells born at distinct times, thus significantly increasing the resolution of the cell fate analysis (20, 41, 88, 89) (Fig. 2*A*). Moreover, double or triple S-phase labeling offers unmatched flexibility in experimental design and reduces the number of experimental groups, thus drastically facilitating the workflow.

### “Window” labeling for tracking cells after terminal mitosis

One of the major disadvantages of the pulse-chase labeling scheme is that this type of labeling does not enable judgment about what happens to the labeled cells during the chase period. Pulse-chase labeling does not reveal whether the labeled cells undergo additional rounds of division or exit the cell cycle soon after having been labeled. Although there were attempts to discriminate between heavily labeled cells that completed their division and faintly labeled cells that underwent additional rounds of division, these data are considered doubtful. “Window” labeling overcomes this problem. It is a variation of double S-phase labeling and combines “pulse” and “cumulative” labeling (17). In this labeling scheme, the first nucleotide analogue is administrated by continuous delivery that lasts no longer than average duration of a single cell cycle in an examined cell population (Fig. 2*B*). Immediately after completion of the first nucleotide analogue delivery, the second nucleotide analogue is continuously delivered throughout the rest of the experiment. Cells that incorporated the first label, but not the second, underwent terminal mitosis within the time interval between the beginning of first label administration and the onset of second label exposure (Fig. 2*B*). Thus, this labeling scheme enables the fate of those cells that underwent terminal mitosis at a specific time window to be traced. This approach offers unmatched opportunities for tracking differentiation, migration, and survival of postmitotic stem cell progeny.

### Label-retaining assay

Many subsets of somatic stem cells within adult mammalian organisms are characterized by a prolonged mitotically inactive state (G_0_-phase). This state, frequently referred to as quiescence or dormancy, is reversible, and quiescent stem cells intermittently enter the cell cycle. Such behavior is characteristic for hematopoietic, muscular, and hair follicle stem cells (91, 92, 93, 94, 95). It has also been demonstrated that the intestine, the tissue with the most intensive self-renewal, harbors a subset of the so called “+4 stem cells,” which undergo infrequent divisions to fuel a pool of rapidly dividing stem cells in intestinal crypts (95, 96, 97). Such infrequently dividing somatic stem cells have been identified using the label-retaining assay. The label-retaining assay resembles pulse-chase labeling, but cells usually undergo marking over prolonged period *via* continuous delivery of a nucleotide analogue instead of pulse labeling (Fig. 2*C*). During the chase period in the absence of the label, the rapidly dividing cells dilute the incorporated label to an undetectable level by repeated cycles of the DNA replication and its distribution between daughter cells. While the rapidly dividing cells dilute the incorporated label, the infrequently dividing cells retain the label over a long period. Therefore, this population of cells is referred as label-retaining cells (LRCs). Although LRCs can be observed when pulse-chase labeling has been conducted, a prolonged labeling instead of pulse labeling is necessary to mark as many as possible infrequently dividing stem cells, which usually represent a minor population.

## Labeling schemes and respective readouts for revealing key parameters of cell cycle kinetics

### Preconditions for applying labeling schemes

The labeling schemes described below allow for determination of key parameters of cell cycle kinetics at the population level. Therefore, application of these labeling schemes necessitates several preliminary assumptions important for valid interpretation of the results. First, the examined proliferative cell population must be homogeneous in terms of the S-phase and entire cell cycle durations. Second, the cells in the population must be randomly distributed throughout all phases of the cell cycle, *i.e.*, the cell population must be asynchronous. Third, the proliferative cell population must be in a steady-state growth phase, *i.e.*, the number of cells that are currently in the cell cycle does not change significantly during the experiment. Fourth, the proliferative cell population must be spatially confined, *i.e.*, cells should not extensively migrate outside of the site of their initial location. Fifth, cell death events are rare and can be neglected. The last two requirements are necessary for the valid quantification of labeled cells.

### Cumulative labeling for determining the cell cycle and S-phase durations

**Figure 3:**
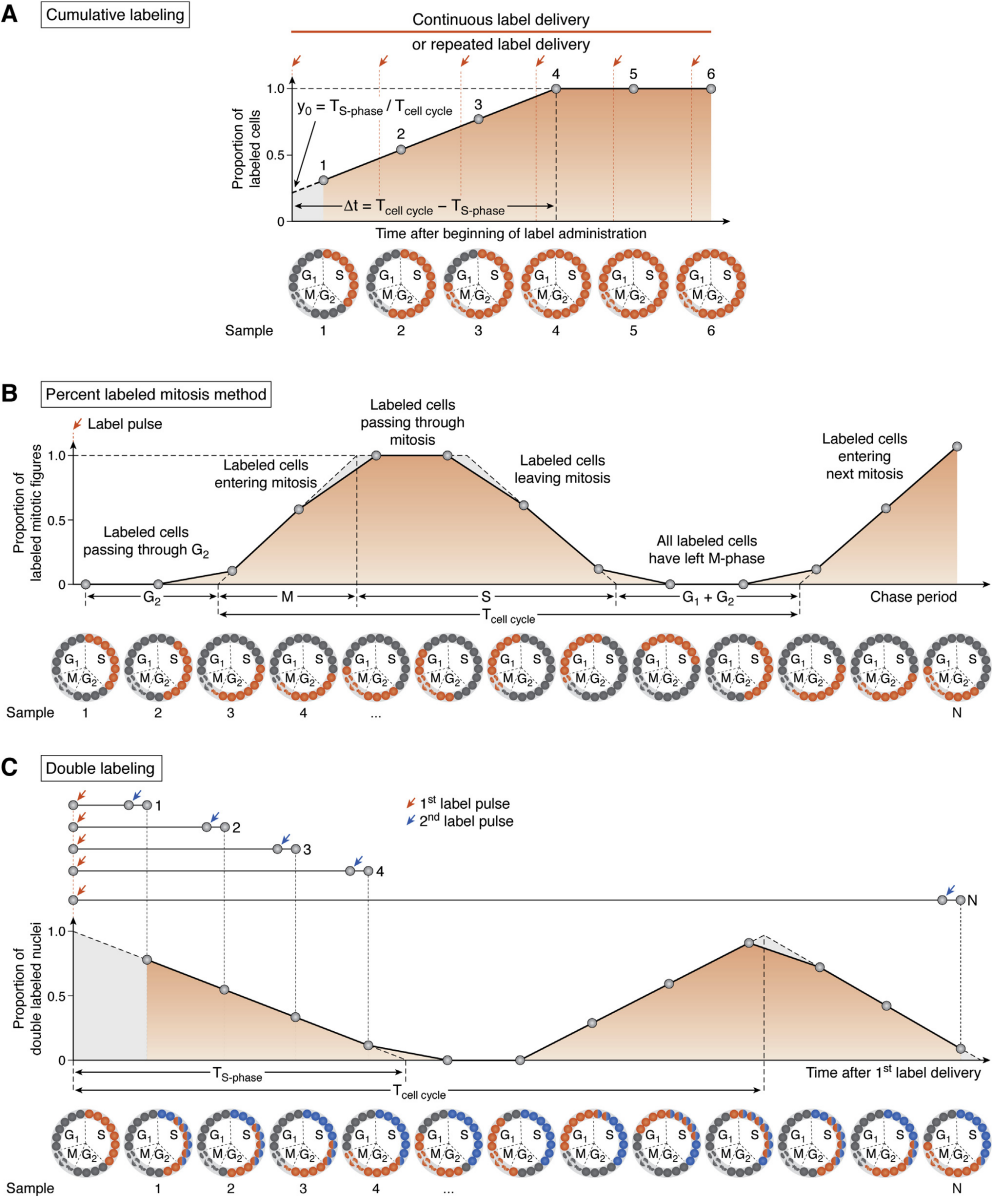
**Labeling schemes for revealing progression through the cell cycle and determining S-phase duration and cell cycle length.***A*, cumulative labeling (adapted from Nowakowski *et al.* (101)). *B*, percent labeled mitoses method (adapted from Cai *et al.* (100)). *C*, double labeling (adapted from Encinas *et al.* (64)). G_1_, S, G_2_, and M, phases of the cell cycle; T_cell cycle_, cell cycle duration; T_S-phase_, S-phase duration; Δt, an intercept between the linear regression line of the slope and the plateau, Δt = T_cell cycle_−T_S-phase_; y_0_, an intercept between the y-axis and the continuation of the slope, y_0_ = T_S-phase_/T_cell cycle_.

Cumulative labeling is extensively applied for determining key parameters of the cell cycle and is conducted *via* either continuous nucleotide analogue delivery (usually for *in vitro* labeling) (98, 99) or repeated pulse labeling (for *in vivo* labeling) (100, 101, 102, 103, 104, 105, 106, 107, 108, 109, 110). Cumulative labeling is aimed to mark all cells of the proliferative population and to track the kinetics of label incorporation into cells passing the cell cycle (Fig. 3*A*). In this labeling scheme, a nucleotide analogue is available for incorporation into replicating DNA throughout the duration of the experiment. Subsets of specimens are periodically collected for counting the cells that have incorporated the label. Then, the dependence of cell counts from the time intervals between the beginning of label delivery and collection of the specimens for analysis is calculated. This dependence linearly increases with the lengthening of time intervals between the beginning of label delivery and collection of the specimens for analysis. The increase occurs because some cells have left S-phase, remaining labeled, while other cells have entered S-phase and therefore, have *de novo* incorporated the label. However, the cells that have already incorporated the label at the beginning of nucleotide analogue exposure eventually re-enter the S-phase. Therefore, all cells circulating in the cell cycle have been marked with a nucleotide analogue, and the number of labeled cells stops increasing, reaching a plateau (maximal value). An intercept between the linear regression lines of the slope and the plateau enables determination of the time interval (Δt) equal to T_cell cycle_−T_S-phase_. If we normalize cell counts to the maximal value, an intercept between the y-axis and the continuation of the slope (y_0_) enables estimation of the ratio T_S-phase_/T_cell cycle_. This enables creating an equation system:

(1) {Δt=Tcellcycle−TS-phase,y0=TS-phaseTcellcycle,

where Δt and y_0_ are the parameters determined in Figure 3*A*, T_S-phase_ is the S-phase duration, and T_cell cycle_ is the cell cycle duration. Therefore, solving the equation system (Equation 1) enables determination of the S-phase duration and the cell cycle duration.

Cumulative labeling has extended the analysis of cell proliferation beyond the routine determination of the dividing cell numbers and allowed for measurement of cell cycle kinetics in diverse experimental situations (106, 107, 108, 109, 110). It should be noted that the linear increase of the dependence indicates that the proliferative cell population satisfies the preliminary assumptions. If the increase in the dependence is not linear, the examined proliferative cell population is either not homogeneous or is not asynchronous, or both and the determination of key cell cycle parameters using cumulative labeling is impossible.

### Percent labeled mitoses method for comprehensive analysis of the cell cycle kinetics

It may seem that the pulse-chase labeling method mentioned above provides very limited information regarding cell division; however, its variation, referred to as the percent labeled mitoses method, enables measurement of the duration of each cell cycle phase (Fig. 3*B*) (100, 110). Pulse labeling marks a cohort of cells that are in the S-phase. Progressing through the cell cycle, this cohort enters mitosis. The percent of labeled mitotic figures starts growing, reaching a plateau at 100%. The percent of labeled mitotic figures will remain 100% until all labeled cells pass. This percentage starts decreasing when unlabeled cells reach the M-phase. The percent of labeled mitotic figures equals 0% again when all labeled cells complete mitotic division. The next rise of the percent of labeled mitotic figures occurs when labeled cells enter mitosis again. The time interval between pulse labeling and the appearance of the first labeled mitotic figure corresponds the duration of the G_2_-phase. The time interval when the percent of labeled mitotic figures grows from 0 to 100% is the duration of the M-phase. The time interval between time points where the percent of labeled mitotic figures initially increases to 100% and then reduces to 0% again is the duration of the S-phase. The time interval between two subsequent rises of the percent of labeled mitotic figures corresponds to the entire duration of the cell cycle.

Similarly, the distribution of cycling cells between cell cycle phases can be determined by pulse labeling combined with the detection of the proliferating cell markers Ki67 or the proliferating cell nuclear antigen (PCNA) and mitotic marker, phosphohistone H3 (108, 111).

### Double labeling for revealing progression of cells through the cell cycle

Another approach allowing for progression of cells through the cell cycle to be traced is based on the use of two labels delivered with a time interval (Fig. 3*C*) (14, 20, 64). The proportion of cells that have incorporated both labels declines progressively as the interval between the labels lengthens because the cells marked with the first label leave the S-phase, and unlabeled cells enter the S-phase and become marked with the second label only. The intercept of the declination line with the time axis (Fig. 3*C*) reveals the time point when all cells marked with the first label exit the S-phase, giving an estimation of the S-phase duration. When we further lengthen the time interval between the two labels, the proportion of the double labeled cells starts growing, reflecting the entry of cells that have incorporated the first label into the next S-phase. Then, the proportion declines again. The time point when the peak is reached corresponds to the time interval between two consecutive S-phases or, in other words, the cell cycle duration.

If the S-phase and cell cycle durations are known approximately for a given proliferative population, simplified double labeling can be applied for estimation of the S-phase and cell cycle lengths (112, 113). In this labeling scheme, one group of animals receive two labels separated by a time interval shorter than the S-phase duration, and subsequent cell quantifications enable the calculation of the S-phase length. Another group of animals receive two labels separated by a time interval longer than the difference between the cell cycle length and the S-phase duration, and subsequent cell quantifications enable the calculation of the cell cycle length. This approach can be applied when we need to test whether any physiological or pathological stimulus alters the cell cycle parameters of a given population of proliferating cells (113, 114, 115, 116).

### Double and triple S-phase labeling schemes for discrimination between proliferating and quiescent subpopulations of daughter cells

**Figure 4:**
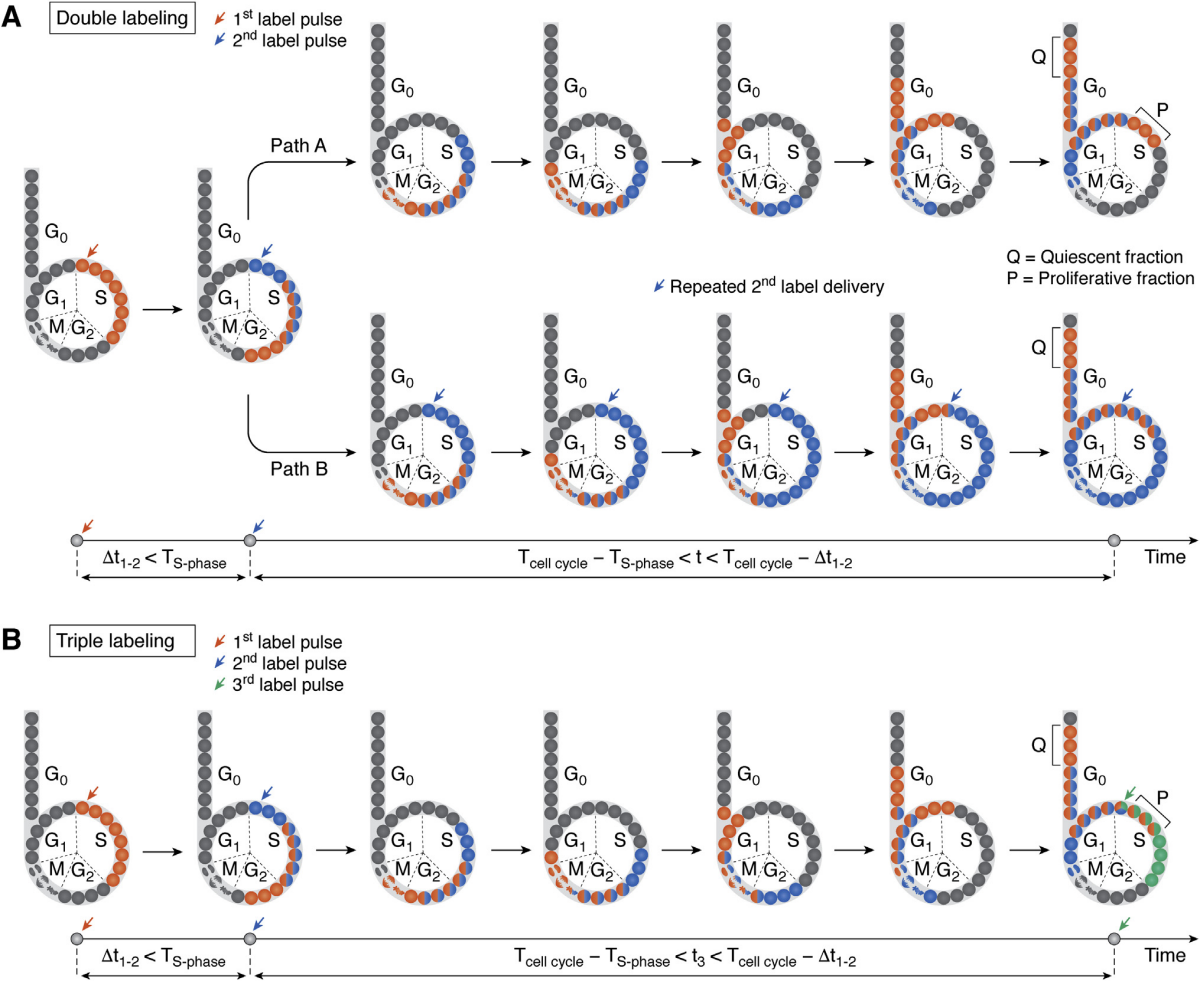
**Labeling schemes for the discrimination between proliferating and quiescent subpopulations of daughter cells.***A*, double labeling in separate experimental groups (adapted from Takahashi *et al.* (16, 117)). *B*, triple labeling in the same experimental group (adapted from Podgorny *et al.* (41)). G_1_, S, G_2_, and M, phases of the cell cycle; P, proliferative fraction; Q, quiescent fraction; t, the time interval during which the second label is repeatedly delivered; t_3_, the time point when the third label is delivered; T_cell cycle_, cell cycle length; T_S-phase_, S-phase duration; Δt_1−2_, a time interval between the first and the second label pulses.

Generally, there are three possible fates of daughter cells after mitosis of stem and progenitor cells. Both daughter cells remain in the cell cycle, both daughter cells exit the cell cycle, or one cell re-enters the cell cycle and the other becomes mitotically quiescent. The ratio between re-entering and exiting the cell cycle defines the kinetics of cyto- and histogenesis during normal development or physiological tissue renewal in adults. If the fraction of daughter cells that re-enter the cell cycle exceeds that of daughter cells leaving the cell cycle, the proliferative population is expanding; otherwise the proliferative population is becoming exhausted. Determination of these fractions is critical for creating kinetic models of cyto- and histogenesis (14, 16, 117). Takahashi *et al*. (16) have suggested a double labeling scheme to trace a limited cohort of cells after mitosis in terms of their proliferative fates (Fig. 4*A*). In this labeling scheme, two pulse labels are delivered at a time interval (Δt_1−2_) shorter than the average S-phase duration in the cell population studied. The cells that incorporate the first label leave the S-phase prior to delivery of the second label, becoming a cohort of labeled cells with known position within the cell cycle. Then, one group of samples (animals or cell cultures) is collected for analysis at a time point (t) after the second label delivery (Path A in Fig. 4*A*) that meets the following criteria:

(2) $${\text{T}}_{\text{cell cycle}}-{\text{T}}_{\text{S}-\text{phase}}<\text{t}<{\text{T}}_{\text{cell cycle}}-\Delta {\text{t}}_{1-2},$$

where T_S-phase_ is the duration of the S-phase, T_cell cycle_ is the cell cycle duration, and Δt_1−2_ is a time interval between delivery of two labels. In parallel, another experimental group receives additional deliveries of the second label followed by analysis at the same time point (Path B in Fig. 4*A*). The cells that have incorporated the first label but not the second one pass mitosis and a portion of them enter the S-phase of the next cell cycle within the time interval determined by Equation 2. In the first experimental group (Path A in Fig. 4*A*), the population of cells that have incorporated the first label includes both the proliferative and quiescent subpopulation of daughter cells. Due to the availability of the second label during the time interval determined by Equation 2 in the second experimental group (Path B in Fig. 4*A*), cells that have initially incorporated the first label become double labeled when they enter the subsequent S-phase. Therefore, in this group, cells that incorporated only the first label belong to the subpopulation of mitotically quiescent progenies. In this case, the quiescent fraction (Q) is calculated as the ratio:

(3) Q=NPathB1NPathA1,

where ${\text{N}}_{\text{PathB}}^{1}$ is the number of cells with the first label only in the second group (Path B in Fig. 4*A*), and ${\text{N}}_{\text{PathA}}^{1}$ is the number of cells with the first label only in the first group (Path A in Fig. 4*A*). The proliferative fraction (P) is calculated as:

(4) P=1−Q,

where Q is determined by Equation 3.

The suggested methodology, however, requires analysis of two experimental groups. Recently, we presented an improved protocol for the determination of cell fractions that re-enter or exit the cell cycle (Fig. 4*B*) (41). This protocol is based on triple labeling and does not require separate experimental groups. Similar to the previous labeling scheme, a cohort of cells with a known position in the cell cycle is highlighted by two pulse labels separated by a time interval (Δt_1−2_) shorter than the average S-phase duration. Then, the third label is delivered at a time point (t_3_) after delivery of the second label that meets the following criteria:

(5) $${\text{T}}_{\text{cell cycle}}-{\text{T}}_{\text{S}-\text{phase}}<{\text{t}}_{3}<{\text{T}}_{\text{cell cycle}}-\;\Delta {\text{t}}_{1-2},$$

where T_S-phase_ is the duration of the S-phase, T_cell cycle_ is the cell cycle duration, and Δt_1−2_ is a time interval between delivery of two labels (the first and the second). In this labeling scheme, the cohort of cells with the first label only is the quiescent fraction Q, whereas the cohort of cells that incorporated the first and the third label but not the second one is the proliferative fraction P.

Notably, both methodologies necessitate determination of the S-phase duration and the cell cycle length.

## Caveats to consider when applying labeling with modified nucleotides

### Remarks for application of pulse chase and cumulative labeling

Pulse-chase labeling with a short chase period is the simplest labeling scheme that is commonly used to address the question of whether an examined stimulus elicits changes in proliferative activity of a certain cell population *in vivo* or *in vitro* (proliferation assay). Comparison of labeled cell numbers between experimental (exposed to a stimulus) and control (nonexposed to a stimulus) groups is mainly interpreted in terms of increased/decreased cell proliferation. However, despite its simplicity, pulse-chase labeling can produce compromised results in certain cases. For instance, it might be assumed that, for a certain cell population, a stimulus elicits changes in duration of the individual cell cycle phases with no effect on overall number of cells circulating in the cell cycle. In this case, proliferation assay performed using pulse-chase labeling will reveal an increase/a decrease in the number of labeled cells that is proportional to a stimulus-induced increase/decrease in the ratio of the S-phase duration to the cell cycle length. Therefore, the observed differences in labeled cell numbers in experimental and control groups cannot be interpreted in terms of changes in proliferative activity. This hypothetical situation demonstrates ambiguousness of data interpretation after pulse-chase labeling and imposes necessity on conducting proliferation assay in a combination with the evaluation of the cell cycle parameters to gain valid labeling results.

Cumulative labeling *via* the repeated pulse delivery of a label is frequently used to mark all cells in the proliferative population or a large cohort of dividing cells to enhance accuracy of quantitative readouts. In most studies, a choice of time intervals between the repeated pulse deliveries of a label is random and reasonless. To perform cumulative labeling rationally, preliminary estimation of the S-phase duration and the cell cycle length is necessary. Evidently, there is no reason to deliver pulse labels at time intervals shorter than the S-phase duration because some cells that have already incorporated a label are remaining in the S-phase and, therefore, receive additional doses of a label that can exert the cytotoxic effect (see below) in labeled cells. Hence, time intervals near the S-phase duration enable labeling distinct cohorts of cells within the same proliferative population, and, moreover, labeled cells in these cohorts receive a single dose of a label. For instance, neural progenitors in the hippocampal dentate gyrus of the adult mouse brain represent a relatively homogeneous cell population with the approximate S-phase duration of 10 to 13 h and the approximate cell cycle length of 23 to 28 h (64, 112, 113). Hence, two pulse label deliveries at the 12 h interval will enable marking almost 90% of the proliferative population with low risk of implementation of two label doses into the same cells.

These speculations indicate that application of any labeling scheme and interpretation of labeling results should be based on the preliminary determined or at least hypothetical cell cycle kinetics of the examined cell population. Otherwise, compromised results or misused labeling can be expected.

### Bioavailability of modified nucleotides

When we label cultured cells with a thymidine analogue in a Petri dish, we are able to precisely control the time interval during which the label incorporates into newly synthesized DNA. The label incorporation can be interrupted by withdrawal of the thymidine analogue *via* simple washing. Optionally, cells can be washed followed by incubation with culture medium supplemented with regular thymidine. Regular thymidine competes with the residual thymidine analogue, thus blocking its incorporation into the replicating DNA. Therefore, loading of the label into cell culture can be as short as 1 min, if necessary. The same scenario of interruption of label incorporation can be applied to marine invertebrate species. In sharp contrast to cultured cells and marine invertebrates, incorporation of a thymidine analogue into the replicating DNA after a single pulse delivery (*via* either an intraperitoneal injection or an intravenous injection) into vertebrate species cannot be precisely controlled and is determined by the bioavailability of the label. Bioavailability is an integrative concept that describes both the fraction of a drug that reaches its site of action and the rate at which the drug becomes available at its site of action. Here, we will primarily refer to bioavailability time, which is determined as the time interval during which the thymidine analogue is completely metabolized by an organism after a single pulse labeling (an intraperitoneal injection or an intravenous injection). Beyond this time interval, the thymidine analogue is no longer available for incorporation into replicating DNA. The bioavailability time of a thymidine analogue depends on the dose injected, the species used, the stage development of the organism, diffusion into the blood circulatory system from the peritoneal cavity or digestive system (if a thymidine analogue is delivered nonintravenously), distribution with the blood stream, penetration into tissues (in particular, penetration through various blood-tissue barriers), and active pyrimidine transport in the cells.

^3^H-thymidine, which is a counterpart to natural thymidine, has been found to rapidly eliminate from the blood plasma after intravenous injection in rodents, monkeys, and humans (118, 119, 120). This elimination occurs *via* two phases: (i) a rapid phase with an approximate half-time of 1 min and (ii) a slow phase with an approximate half-time varying from 10 to 20 min depending on the species (118, 119, 120). Minute amounts of radiolabeled thymidine remained detectable in the blood plasma up to 60 min after intravenous injection. Autoradiographic analysis of blood cell concentrates or tumor tissue sections revealed that the bulk of ^3^H-thymidine was incorporated into DNA within 15 to 25 min after a pulse dose and that the uptake of ^3^H-thymidine achieved a plateau at approximately 40 to 60 min (119, 120).

Only a portion of the injected BrdU incorporates into the replicating DNA after a single intravenous injection. The bulk of the injected BrdU is rapidly degraded with the formation of bromouracil and bromide ions (121, 122). Blood serum enzymes are likely to convert BrdU into bromouracil (122), and the liver plays a major role in dehalogenation of BrdU (121). The bioavailability time of BrdU was determined by distinct approaches, and the current estimation varies from 15 min to 2 h after a single pulse labeling in rodents (14, 40, 121, 123, 124, 125). It seems that indirect methods that relied primarily on the quantification of labeled cells underestimated the bioavailability time, giving an estimation of 30 min or even less (14, 40, 123). Direct measurements of radiolabeled BrdU revealed that BrdU is no longer available for incorporation into DNA within a time interval between 1 h and 2 h after a single dose delivery in rodents (121, 124). Similarly, the bioavailability time of BrdU estimated by measuring BrdU content by high-performance liquid chromatography assay in blood serum after an intravenous injection in dogs was approximately 2 h (126). When radiolabeled BrdU or ^3^H-thymidine was intraperitoneally injected into pregnant mice, the half-life of the nucleotide analogues in the embryos was found to be longer than in various maternal tissues, 60 to 80 min *versus* 30 min, respectively. Thus, the longer bioavailability time of the thymidine analogues must be taken into account when labeling dividing cells in embryos of mammalian species.

The bioavailability time for the rest of the nucleotide analogues used for labeling replicating DNA has not yet been evaluated. Our indirect observations on the progression of hippocampal neural progenitors through the cell cycle using triple S-phase labeling suggest that the bioavailability time of CldU, IdU, and EdU is shorter than 2 h (41). We sequentially injected mice with the three labels with 2-h intervals and counted the cells that incorporated the labels. The observed labeling pattern (numbers of cells with definite combinations of the labels) was consistent with the hypothetical linear progression of neural progenitors through the cell cycle (see Fig. 2, *A*–*C* in (41)). We repeated the experiment, injecting labels in a different order, and observed that the labeling pattern was independent of the order of label delivery (unpublished data). If there was at least one nucleotide analogue with a bioavailability time over 2 h, the observed labeling pattern should have differed from the expected pattern.

Bioavailability of a nucleotide analogue can be neglected in the following *in vivo* labeling situations: (i) pulse-chase labeling where the chase period is quite long (longer than a day) and (ii) prolonged (longer that a day) cumulative labeling when a thymidine analogue is delivered with drinking water, by repeated injections, or *via* an implanted osmotic pump. However, bioavailability must be always taken into consideration when the S-phase or cell cycle kinetics is analyzed using pulse-chase labeling (100, 110), cumulative (101), double (14), or triple (41) labeling. Inadequate labeling may occur when the time intervals between label pulses or between label pulses and euthanasia do not exceed the bioavailability time of thymidine analogues.

### Cytotoxicity of modified nucleotides

Cytotoxicity of modified nucleotides is a serious pitfall for the study of cell proliferation and retrospective birth dating of cells. Toxic manifestation of modified nucleotides depends on the dose used, administration technique (single, multiple, or continuous administration), and time elapsed after treatment.

Toxic effects of the most widely used nucleotide analogue BrdU were examined in various experimental situations. BrdU was initially demonstrated to affect growth of mammalian cancer cells (127). More recently, incorporation of BrdU has been shown to inhibit cancer cell proliferation *in vitro* and delay tumor progression *in vivo* (128). It has been demonstrated that transient exposure of various cancer cell lines to BrdU at a dose of 1 μM or higher suppresses the rate of cell expansion. This effect becomes detectable within several days of exposure. However, BrdU-treated cells do not die. Instead, they alter the timing of the cell cycle, primarily increasing the duration of the G_1_ and G_2_ phases, and upregulate senescent-associated proteins. Both intraperitoneal injections of BrdU (six pulse doses 300 mg/kg within 2 days) and oral delivery of BrdU (0.8 mg/ml, for 7 days) suppressed progression of grafted tumors in rats (128). BrdU, when injected into pregnant mice at a dose 500 mg/kg, was shown to cause multiple abnormalities of brain development in embryos (129). This dose was higher than the saturating dose (150 mg/kg) determined for mice (61). A single intraperitoneal injection of BrdU at a dose 50 mg/kg had no apparent toxic effect on the developing cortex in rats (130). A single intravenous injection of BrdU at 50 mg/kg caused abnormalities in the numbers and distribution of labeled cells in the developing cerebral cortex of macaque monkeys (131). This contradiction in the evaluations of toxic effects of BrdU in rodents and monkeys may be explained by the dramatic differences in brain sizes and rates of cortical development (131). In another study, pregnant rats received five to six intraperitoneal injections of BrdU at a dose 12 to 20 mg/kg at 8-h intervals (132). This exposure reduced litter size and body weight, increased mortality of the offspring, and caused multiple defects of the cerebellum in the adult progenies (132). BrdU was also found to induce senescence-like processes in a variety of cells (133, 134) and increase the risk of sister-chromatid exchange and the induction of specific-locus mutations (135, 136). Both CldU and IdU also exhibit cytotoxicity (137, 138). However, their toxic effects were primarily studied in the context of anticancer therapy and have not been yet evaluated in the context of labeling dividing cells. Therefore, it is difficult to judge whether they are more or less toxic than BrdU.

Virtually all studies that have addressed adverse effects of EdU indicate that this substance is highly toxic. The toxic effect of EdU was found to be more pronounced than the toxic effect caused by an equimolar dose of BrdU in various cells both *in vitro* and *in vivo* (31, 102). While 10 μM BrdU reduced the rate of cell expansion, the same dose of EdU evoked progressive cell death in various cell lines (31). The adverse effects of EdU, such as cell cycle arrest and cell death, are primarily linked to its genotoxicity (139). Additionally, EdU has been also demonstrated to inhibit several enzymes involved in nucleoside metabolism (139, 140, 141). This may underlie the adverse effects of EdU such as slowdown of cell cycle progression (139) and suppression of BrdU incorporation into the DNA when BrdU is administrated immediately after EdU exposure (25). Interestingly, in most cases, the toxic effects of BrdU and EdU become noticeable if the chase period after single exposure exceeds the duration of the cell cycle (31, 102, 128).

To evaluate the cytotoxicity of the other chemically detectable nucleotide analogues, F-ara-EdU, AmdU, and VdU, cellular respiration was assayed in a variety of cell lines (24, 25, 26). Generally, all these nucleotide analogues were found to be less toxic than EdU (24, 25, 26). F-ara-EdU was also less toxic than BrdU, whereas VdU and AmdU were more toxic than BrdU. Therefore, nucleotide analogues can be evaluated in terms of their relative cytotoxicity (Table 1).

In regard to the triple labeling method, EdU might always be considered to be used as the last label due to it having the highest toxicity. The chase period after EdU treatment should not exceed the duration of the cell cycle. Saturating doses of the thymidine analogues may be recommended if the S-phase or cell cycle progression is studied because this type of experiments does not usually require extended chase periods. Doses below the saturating dose may be used if the retrospective birth dating of cells is investigated using extended chase periods.

### Stability of modified nucleotides within DNA: a critical remark for the long-term labeling

Tracing the fate of dividing stem cells and their progeny in diverse *in vitro* and *in vivo* systems relies on the ability of a nucleotide analogue to remain within the DNA for a prolonged period. Dilution of the label incorporated into the DNA to undetectable levels after pulse labeling may occur *via* repeated cycles of DNA replication in frequently dividing cells. If cells do not pass too many divisions after pulse labeling, they are believed to retain the label for an unlimited period of time. In particular, BrdU incorporated into the DNA of adult-born neurons in the human brain was detected more than 2 years after a single intravenous infusion (90). Similarly, BrdU-positive Purkinje cells were observed in 2-year-old rats after transient treatment of their mothers with BrdU during pregnancy (132). However, BrdU and IdU were found to undergo dehalogenation when integrated into the DNA of cultured cells (142). The uracil residues remaining after dehalogenation are removed from DNA by uracil glycosylase (142, 143). This process is thought to underlie the continuous loss of the label over a prolonged period of time. Therefore, the decrease of the number of labeled cells over a prolonged period must be interpreted with caution because this decrease could originate from the loss of the label *via* its removal rather than from the death of labeled cells. It has been also demonstrated that IdU is excited from DNA more slowly than BrdU (142). Therefore, the differences in the rate of the excision from DNA among various nucleotide analogues should be taken into account when performing multiple S-phase labeling. In sum, these observations reveal the importance of the pre-evaluation of label behavior over a prolonged period if long-term experiments with quantification of labeled cells are planned. Excision of chemically detectable nucleotide analogues (EdU, F-ara-EdU, AmdU, and VdU) from DNA has not yet been demonstrated.

### Are nucleotide analogues capable of marking DNA repair?

This is a crucial question regarding the application of modified nucleotides for labeling dividing stem cells because marking DNA repair may lead to false-positive results that, in turn, call the applicability of the method into question. One of the more thorough studies of this issue was conducted on mouse and human pancreatic β-cells (144). To clarify the issue, the authors evaluated nuclear colocalization of BrdU and γ-phosphorylated H2A histone family member X (γH2AX), a DNA damage and repair marker. They performed a series of experiments under various conditions to explain the appearance of β-cells with double-labeled nuclei. One of them was designed to assess the effect of DNA damage on BrdU incorporation directly. In this experiment, mouse and human β-cells were exposed to a sublethal dose of mitomycin C or to UV irradiation. The first type of exposure evoked damage by DNA cross-linking, and the second led to the appearance of pyrimidine dimers from adjacent thymine bases. Neither mouse nor human β-cells showed an increase in BrdU incorporation in response to either type of DNA damaging exposure. Instead, the authors found that the DNA damage suppressed BrdU incorporation. Unlike some of the other experimental paradigms applied in the article, the frequency of BrdU and γH2AX colabeling was not increased in comparison as predicted by random chance level, where BrdU and γH2AX colabeling happens as unrelated events. These observations have led to the conclusion that the appearance of the γH2AX and BrdU colabeling is related to events associated with cell cycle progression and cell division rather than DNA repair. Similarly, density-arrested normal diploid fibroblasts were used to determine the contribution of DNA repair to BrdU labeling (145). It was expected that the DNA repair induced by radiation exposure to nonmitotic cells would lead to an increase BrdU incorporation. Despite the appearance of double-strand breaks, no enhancement of BrdU labeling occurred. In another study, quantitative evaluation of ^15^N-thymidine incorporation into DNA using multi-isotope imaging mass spectrometry (see below) revealed that the labeling level of dividing cardiac fibroblasts was two orders of magnitude higher than the labeling level of fibroblasts subjected to hydrogen peroxide, an oxidative stress agent (146). The authors attribute this to the negligible number of bases that can be replaced during repair compared with the number of bases in the entire genome.

Taken together, these observations indicate that, even when thymidine analogues incorporate into DNA during repair, this incorporation remains at a level undetectable by conventional detection methods. This validates application of modified nucleotides for tracking dividing stem cells.

## The most recent advances in the detection of replicating DNA

### The new generation of chemically detectable nucleotide analogues

F-ara-EdU, which belongs to a small family of 2′-arabino-modified 5-ethynyluridine derivatives, and AmdU is an azide-modified nucleoside, are detected *via* the azide–alkyne click reaction and are less toxic than EdU (24, 25). Successful application of F-ara-EdU for labeling dividing cells *in vivo* was confirmed in zebrafish and planarians (24, 147, 148). The detection of AmdU was demonstrated as not interfering with the identification of EdU and BrdU, thus allowing for triple S-phase labeling (25). AmdU incorporated into DNA can also be detected *via* copper-free, strain-promoted cycloadditions with cyclo-octynes where a bicyclo[6.1.0]nonyne-modified dye is used as a fluorescent probe. This approach allows for detection of AmdU-labeled DNA in live cells. However, permeabilization of cell membranes is necessary for bicyclo[6.1.0]nonyne-modified dye penetration into live cells and leads eventually to cell death. Therefore, utility of this approach is illusive.

VdU incorporated into DNA is detected by tetramethylrhodamine–tetrazine conjugate binding *via* an alkene–tetrazine ligation reaction (see Scheme 1b and Scheme S1 in (26)). Importantly, in contrast to other “clickable” nucleotide analogues, the detection of VdU necessitates DNA denaturation by hydrochloric acid (26). VdU was found to be less toxic than EdU. However, to achieve the same labeling and detection efficiency as a 10 μM EdU dose, a 30 μM dose of VdU is necessary. Because the detection of VdU does not overlap with the Cu(I)-catalyzed [3 + 2] cycloaddition click reaction or interfere with BrdU identification, the set of nucleotide analogues, VdU, F-ara-EdU, and BrdU, is available for triple S-phase labeling (26).

The “clickable” nucleotide analogues offer the following advances for labeling replicating DNA and tracing dividing stem cells. First, quadruple, at least, S-phase labeling of dividing cells is hypothetically possible by combining the halogenated nucleotide analogue BrdU with the nucleotide analogues EdU (F-ara-EdU), AmdU, and VdU. It is also hypothetically possible to combine the “clickable” nucleotide analogues with CldU and IdU, thus allowing for the penta-label marking of replicating DNA in one sample. However, the question on whether the antibodies recognizing CldU and IdU cross-react to the nucleotide analogues F-ara-EdU, AmdU, and VdU has not yet been tested. Increasing the number of nucleotide analogous available for the concurrent labeling of the replicating DNA will (i) facilitate multiparametric examination of stem cell proliferative behavior (proliferative activity and entering, re-entering and exiting the cell cycle) *via* the combination of several labeling schemes in one sample, (ii) increase the resolution of cell fate tracing in terms of the number of cell generations that can be simultaneously identified and in terms of timing of cell generation, and (iii) gain accuracy in determination of the S-phase and cell cycle durations. Second, “clickable” nucleotide analogues enable dividing stem cells and their progeny to be traced in 3D in whole mount preparations (149). Although protocols for staining whole mount preparations with antibodies are available (150, 151, 152), the detection of halogenated nucleotide analogues by antibodies in whole mount preparations is hampered by the necessity of the exposure to hydrochloric acid for DNA denaturation that causes significant shrinking of the tissue sample. “Clickable” nucleotide analogues lack this disadvantage. Moreover, the detection of “clickable” nucleotide analogues by small fluorescent dyes instead of large antibodies provides more uniform staining throughout a whole mount preparation.

However, most “clickable” nucleotide analogues are not widely used in stem cell research. The major reason that application of these nucleotide analogues is still limited is that they are extremely expensive, especially for *in vivo* studies where large amounts of substances are necessary.

### Chemical detection of BrdU

A recent report has offered a revolutionary strategy for the identification of BrdU incorporated into cellular DNA (27). This novel strategy relies on chemical detection of cellular BrdU instead of traditional identification *via* antibody application. Chemical detection of BrdU is conducted *via* an optimized Suzuki–Miyaura reaction. A Suzuki–Miyaura reaction is a cross-coupling reaction between a boronic acid derivate and an organic halide. This cross-coupling is catalyzed by a palladium (0) complex (see Fig. 1*B* in (27)). The optimized Suzuki–Miyaura reaction for the detection of cellular BrdU does not necessitate DNA denaturation. Coupling the fluorescent derivate of boronic acid to cellular BrdU is conducted in two steps. BrdU-labeled cells are initially treated with the DTBPPS/Palladium catalyst system under anaerobic conditions (in argon atmosphere) at 37 °C (the DTBPPS/Palladium catalyst system is prepared by mixing DTBPPS (3-(Di-tert-butylphosphonium)propane sulfonate) and sodium D-isoascorbate in H_2_O in H_2_ atmosphere followed by addition of stock solutions of K_2_PdCl_4_ and nBu_4_N^+^OH^−^; for details, see (27)). While cells are undergoing treatment with the DTBPPS/Palladium catalyst system, stock solutions of fluorescent boronic probe and sodium-L-ascorbate are mixed in argon atmosphere to prepare a fluorescent boronic mixture. Next, the fluorescent boronic mixture is added into the DTBPPS/Palladium catalyst system. Then, the cells are incubated in the whole reaction mixture in a H_2_ atmosphere. Elimination of reactive oxygen from the reaction system in all stages of the protocol is a critical requirement for the successful chemical detection of BrdU. This protocol has been shown to produce bright BrdU staining without nonspecific fluorescent nuclear labeling both *in vitro* and *in vivo*. Chemical detection of BrdU has been found to be as sensitive as traditional detection by anti-BrdU antibodies. The detection of cellular BrdU by the optimized Suzuki–Miyaura reaction does not interfere with the detection of EdU by the click reaction, thus allowing for the discrimination of these nucleotide analogues in double-labeled specimens. Although chemical detection of BrdU seems to be a quite promising method, its application in formaldehyde-fixed cells or tissues has not yet been demonstrated. Formaldehyde fixation may interfere with chemical detection of BrdU, thus confining a range of experimental situations where this method can be applied.

### Multi-isotope imaging mass spectrometry for tracing dividing stem cells labeled with ^15^N-thymidine and ^13^C-thymidine

Multi-isotope imaging mass spectrometry (MIMS) is a tricky methodology that combines labeling with stable isotopes such as ^15^N and ^13^C, ion optics for generation and collection of secondary ions from biological specimens, mass spectrometry, and computational algorithms for analyzing data and creating images (153). To trace replicating DNA using MIMS, cells or organisms are initially treated with ^15^N-thymidine or ^13^C-thymidine. A focused beam of Cs^+^ ions is applied for scanning the biological sample. At each location, Cs^+^ ions sputter a small volume of a matter, thus generating secondary ions. Currents corresponding to ^12^C^−^, ^13^C^−^, ^12^C^14^N^−^, and ^12^C^15^N^−^ ions are recorded to acquire raw MIMS data. Then, the ratios ^12^C^15^N^−^/^12^C^14^N^−^ or ^13^C^−^/^12^C^−^ are compared with the natural abundancies of ^15^N and ^13^C to create images on which the value of each pixel indicates by how much the amount of each isotope exceeds its natural abundance. Hence, bright pixels reveal areas with increased levels of thymidine turnover or, in other words, areas where DNA is undergoing or has undergone replication (146, 153, 154, 155).

There are several advantages of MIMS over traditional approaches for revealing replicating DNA. First, MIMS provides unprecedented planar and depth resolutions, below 50 nm and 1 nm, respectively (153), allowing for 3D reconstruction (156). Second, MIMS is an extremely sensitive methodology, enabling direct measurement of stable isotope content and quantitative data on the turnover of metabolites (153, 154). Third, MIMS can distinguish such combinations of ions as ^12^С^15^N and ^13^С^14^N, thus allowing for the use of multiple labels (up to four: ^15^N-thymidine, ^13^C-thymidine, BrdU (^81^Br), and IdU (^127^I)) within a single biological sample (153, 154, 155). Fourth, isotope-labeled molecules used as probes mimic their natural counterparts and, therefore, do not change cell metabolism and do not exhibit toxicity. Fifth, isotope-labeled molecules can be used for studies in human subjects (154, 157, 158).

Incorporation of isotope-labeled thymidine with subsequent detection of the label by MIMS was applied to test the “immortal strand hypothesis” (154). During DNA replication, the two unwound strands of a double helix serve as templates for building two novel double-stranded helices, which then segregated between daughter cells. According to the “immortal strand hypothesis,” a stem cell when dividing asymmetrically segregates chromosomes so that it always retains the original (immortal) template strand, whereas a committed daughter cell receives the newly synthetized strand. Such nonrandom chromosome segregation was thought to prevent accumulation of harmful mutations resulting from repeated cycles of DNA replication. The “immortal strand hypothesis” predicts the existence of label-retaining cells within populations of actively dividing stem cells. MIMS analysis of incorporation of ^15^N-thymidine into dividing cells in the small intestinal crypts revealed no label-retaining stem cells other than Paneth cells, which are largely quiescent (154). This observation provides evidence against retention of the immortal DNA template strand in stem cells. MIMS combined with labeling by ^15^N-thymidine revealed that cardiomyocyte replacement under physiological and pathological conditions occurs mainly *via* proliferation of pre-existing cardiomyocytes (146). The most recent study using MIMS has demonstrated that the birth of new cardiomyocytes can be stimulated by aerobic exercise (159). These examples show how this approach is valuable for discovering unknown features of stem cell division.

## Concluding remarks

Marking replicating DNA by thymidine analogues is a reliable method for tracing dividing stem cells and their progenies. Seven thymidine analogue species are currently available for labeling after the recent extension of the “clickable” thymidine analogue panel. Resembling natural thymidine, they readily spread throughout the organism *via* the blood circulation system and label dividing cells in all tissues and organs, enabling study of various somatic stem cells *in situ*. Marking replicating DNA is a flexible methodology because distinctions in detection methods enable the combination of thymidine analogues with various assays and usage in diverse experimental situations. The ability to combine temporal discrimination of multiple labels, application of complex labeling schemes, and subsequent quantitative analysis of cells that have incorporated labels, means that labeling replicating DNA by thymidine analogues is a powerful and versatile method for revealing various aspects of the stem cell life cycle, such as modes of stem cell division, proliferative behaviors, maintenance, elimination, and differentiation.

There are several critical points that should always be taken into account when marking replicating DNA by thymidine analogues. First, the dosage of the thymidine analogue and its delivery regimen is a compromise between labeling capacity and harmful effects on dividing cells. Second, the choice of label or a label set depends on the compatibility of the label detection method with other assays that will be concurrently performed. Third, it is always reasonable to know the approximate durations of the S-phase and the cell cycle within the proliferating population being studied. Fourth, whether the proliferating population being studied is homogeneous in terms of the duration of the S-phase and cell cycle and whether the proliferating population being studied is synchronous or asynchronous. Therefore, preliminary examinations of label behavior in each individual experimental situation may be required to avoid misinterpretation of labeling results. These examinations should help the rational design of a labeling scheme and facilitate the subsequent interpretation of observations.

## Conflict of interest

The authors declare that they have no conflicts of interest with the contents of this article.
